# Public health intelligence-ready blueprint for strengthening community resilience: a multi-stakeholder qualitative study in tertiary Chinese hospitals

**DOI:** 10.3389/fpubh.2026.1763588

**Published:** 2026-01-21

**Authors:** Yang Han, Sini Li, Jianling Huang, Xiaojian Jiang

**Affiliations:** 1 School of Civil Engineering, Central South University, Changsha, China; 2 Department of Architecture and Civil Engineering, City University of Hong Kong, Hong Kong, China; 3 Xiangya School of Nursing, Central South University, Changsha, Hunan, China; 4 School of Nursing, Hunan University of Chinese Medicine, Changsha, Hunan, China

**Keywords:** community resilience, data governance, decision support, hospital logistics, information integration, medical equipment management, organizational resilience, predictive maintenance

## Abstract

**Introduction:**

Hospitals must manage complex lifecycles of equipment, consumables, and infrastructure, yet fragmented legacy systems create silos, inefficiencies, and compliance risks that can heighten organizational vulnerability and weaken hospitals’ role as critical infrastructures for community resilience.

**Methods:**

We conducted semi-structured interviews with 16 stakeholders across seven tertiary hospitals in China (administrators; logistics/procurement; facilities/equipment management; clinical representatives) and analyzed transcripts using a hybrid inductive–deductive thematic approach.

**Results:**

Findings converged on seven challenge areas: (1) lifecycle and maintenance bottlenecks; (2) logistics gaps in procurement, inventory, and high-value consumable traceability; (3) limited oversight of resources and energy; (4) system usability issues and data fragmentation; (5) unmet needs for intelligent capabilities (e.g., predictive maintenance; inventory/energy dashboards; emergency workflows; compliance automation); (6) insufficient role-specific training; and (7) security and privacy constraints. Participants emphasized the need for low-friction, interoperable tools that are integrated with Hospital Information Systems (HIS), Hospital Resource Planning (HRP), Office Automation (OA), and Supply–Processing– Distribution (SPD) systems, and capable of providing actionable, role-tuned insights to strengthen coordinated action across professional groups in public health operations.

**Discussion:**

We propose a five-pillar Intelligence-Readiness Framework and 22 design requirements outlining an incremental pathway from foundational interoperability and governance toward intelligence-ready infrastructure operations, to enhance organizational resilience and contribute to broader community preparedness in public health.

## 1 Introduction

Healthcare delivery today depends on the tight coordination of medical devices, consumables, physical facilities, supporting hospital infrastructure, and the clinical–administrative information systems that bind them together. In everyday operations, however, that coordination is fragile. A single disruption, such as an imaging unit awaiting a spare part, a batch of high-value consumables with incomplete traceability, or an undetected utilities anomaly, can propagate across services and degrade throughput, safety, and financial performance. Although such challenges are global, their contours differ by country and by the maturity and integration of hospital information systems (HIS) and operational management models (1). In addition to these immediate operational and financial impacts, such disruptions expose more profound weaknesses in hospitals’ organizational resilience, understood as their capacity to maintain and adapt critical functions under stress. Because tertiary hospitals frequently serve as referral centers and essential nodes in public health systems, fragility in their internal coordination can also compromise community resilience by limiting the continuity and responsiveness of care during large-scale emergencies, where community resilience is understood as the continuity and responsiveness of essential healthcare services for surrounding populations, enabled by resilient hospital operations during disruptive events.

In China’s tertiary hospitals, core information platforms are widely deployed but highly heterogeneous. These typically include HIS (2); Hospital Resource Planning (HRP) or Enterprise Resource Planning (ERP) (3); Office Automation (OA) (4); Supply, Processing, and Distribution (SPD) (5); and Picture Archiving and Communication Systems (PACS) (5) supporting clinical, administrative, logistical, and imaging functions. Introduced incrementally by different vendors and departments, these systems form a fragmented landscape with uneven functionality and limited interoperability. Integration is uneven: data often traverse systems through manual export/import or re-entry rather than via standardized, event-driven interfaces. Asset records in finance modules may be decoupled from maintenance workflows; SPD frequently covers only a subset of materials; and building management systems for energy and utilities are commonly siloed from clinical HIS (6). Unique identifiers for devices, locations, and even encounters across multi-campus settings are not uniformly harmonized, complicating reconciliation and lineage tracking. While standards such as Health Level Seven (HL7) and, more recently, Fast Healthcare Interoperability Resources (FHIR) are known, routine, hospital-wide use remains inconsistent (7). These patterns of fragmentation and *ad hoc* integration can erode hospitals’ ability to anticipate, absorb, and recover from disruptions, thereby weakening key dimensions of organizational resilience in public health. When core platforms are poorly coordinated, it becomes more challenging to sustain essential services during epidemics, mass-casualty incidents, or infrastructure failures in public health, with downstream consequences for the stability and resilience of the communities that depend on these institutions.

The operational and regulatory context further shapes the information ecosystems of Chinese hospitals. National data protection requirements and hard-learned lessons from external service outages drive a strong preference for on-premises or private data center deployments (8). This posture enhances sovereignty and control but can slow adoption of scalable integration services and modern operations tooling unless deliberately addressed (9). Meanwhile, payment reform (e.g., Diagnosis-Related Groups [DRG]/Diagnosis-Intervention Packet [DIP]) and volume-based procurement intensify scrutiny of utilization, cost-effectiveness, and traceability (10). Frontline teams, however, frequently lack unified, trustworthy data on device uptime, workload, and cost to inform procurement, maintenance, and replacement planning (11). Clinical engineering teams are lean in terms of asset footprints and rely extensively on vendor maintenance; backups for critical devices may be scarce, thereby amplifying the operational risk of single-point failures (12). Night-shift and auxiliary staff (e.g., cleaners and porters), who are pivotal for fault detection and reporting, are often underserved in terms of training and system access. In this context, workarounds such as WeChat groups, phone calls, and spreadsheets fill critical gaps but remain brittle, non-auditable, and difficult to scale or standardize. Taken together, these constraints suggest that many Chinese tertiary hospitals operate with limited buffers and constrained adaptive capacity, leaving organizational resilience highly contingent on informal workarounds and individual effort. During system shocks—such as pandemics, extreme weather events, or regional infrastructure disruptions—these latent vulnerabilities may translate into reduced surge capacity and less reliable support for community-level health needs.

Physical facilities and energy oversight present additional challenges. Many hospitals lack comprehensive sub-metering between central and departmental valves, and Heating, ventilation, and air conditioning (HVAC) systems are frequently controlled at coarse granularity (13). During seasonal transitions, simultaneous overcooling and overheating across zones are common, while leaks or surges can go undetected for extended periods. The result is a recognizable pattern of platform sprawl, siloed data, duplicate entry, and manual reconciliation. Users consistently favor solutions that integrate with existing systems, minimize extra clicks, and deliver immediate, role-specific value to clinicians, engineers, logisticians, and managers, while still operating within strict security constraints (14). Inadequate visibility and control over physical facilities and energy systems can further weaken hospitals’ ability to sustain safe and functional environments during periods of stress, such as supply disruptions, extreme weather, or power constraints, thereby affecting both organizational resilience and the reliability of care for local populations.

In contrast, health systems in North America and parts of Europe have, over the past decade, converged on fewer enterprise vendors for electronic health records and related platforms, with regulatory drivers accelerating interoperability (15). The US 21st Century Cures Act, FHIR Application Programming Interfaces (APIs), and the Substitutable Medical Applications and Reusable Technologies (SMART)-on-FHIR app ecosystem have promoted data liquidity and reduced barriers to cross-system integration (16). Many organizations operate centralized command centers for clinical operations and physical facilities, supported by enterprise service buses, event-driven architectures, and 24/7 network/operations centers (17). On the operational technology side, Computerized Maintenance Management Systems (CMMS) and Enterprise Asset Management (EAM) platforms are more commonly integrated with ERP/finance and clinical workflows. Condition-based and reliability-centered maintenance programs leverage telemetry from imaging, operating room (OR) device clusters, and life-support equipment. Real-time location systems (RTLS), typically based on radio-frequency identification (RFID), enhance asset utilization and emergency dispatch, while supply chains are increasingly adopting GS1 (Global Standards One) standards and Unique Device Identification (UDI) for end-to-end traceability, utilizing closed-loop scanning that links items to patients, procedures, and billing (18). Executive dashboards and standardized key performance indicators (KPIs), such as uptime, mean time to repair, maintenance cost, and energy intensity, are widely used for benchmarking and replacement planning (19). These developments have been discussed not only in terms of efficiency gains but also as enablers of more resilient service delivery, by supporting continuous monitoring, rapid reconfiguration of resources, and more agile responses to disruptions at the organizational and system levels.

These international models are not without friction, as cybersecurity threats, vendor lock-in, and complex change management persist, but several differences stand out relative to typical Chinese deployments. Policy-enforced interoperability is stronger; event-driven integration and standardized identifiers are more prevalent; telemetry and RTLS coverage for assets is broader; and CMMS/EAM platforms are more tightly integrated with ERP and clinical systems. A cultural norm of centralized operational monitoring is also more common. Consequently, the emphasis of challenges and needs diverges. Chinese hospitals often prioritize overcoming integration debt across multi-vendor HIS/HRP/OA/SPD landscapes, with “data plumbing” as the first order of business. They face acute pressures from reform-driven cost controls and volume-based procurement, yet data silos and duplicated entries hinder the practical application of analytics. A strong preference for on-premises or private deployments tightens control but raises the bar for in-house integration, security, and operational capabilities (20, 21). Lean clinical engineering staffing and variable levels of digital literacy, especially among night-shift and auxiliary personnel, create pressing needs for low-friction user experiences, scan-to-act workflows, and micro-learning delivered at the point of work (22). Physical facilities integration lags, with limited sub-metering and coarse HVAC control amplifying energy waste and masking anomalies.

These differences do not diminish the shared goals of achieving safer, more efficient, and more resilient operations, but they do influence sequencing and design constraints. In the Chinese context, hospital leaders and frontline teams consistently seek foundational interoperability, streamlined approvals, trustworthy utilization and cost data, and practical tools for maintenance, inventory control, emergency dispatch, compliance automation, and energy oversight, all delivered within stringent security boundaries and without adding cognitive burden (23, 24). Framing these needs in terms of organizational and community resilience highlights that intelligence-ready solutions are not only about automation or optimization, but also about strengthening hospitals’ capacity to prepare for, withstand, and recover from disruptive events while maintaining access to public health for surrounding populations.

A multi-stakeholder perspective was adopted to capture how operational challenges and coping strategies emerge at the intersections of clinical, technical, and administrative work, where coordination failures are most likely to undermine hospital functioning and organizational resilience. Against this backdrop, this study conducted a multi-stakeholder qualitative investigation across several large tertiary hospitals in China. By analyzing the operational characteristics of these hospitals, it comprehensively mapped the “work as done” across the equipment lifecycle, logistics, physical facilities, and information systems; identified recurring pain points and coping strategies; clarified concrete suggestions for advanced integrative capabilities; and translated these insights into a practical, phased blueprint for intelligence-driven hospital operations grounded in frontline realities. The findings provide an empirically grounded view of the infrastructural and operational conditions that hospitals themselves consider necessary for intelligence-ready systems—conditions that are increasingly recognized as essential precursors for strengthening organizational resilience and, indirectly, the capacity of public health systems to support community resilience in future crises.

## 2 Methodology

### 2.1 Design

This research adopted a qualitative descriptive design, utilizing semi-structured interviews and reflexive thematic analysis, to examine day-to-day equipment, logistics, and physical facilities management across both clinical and administrative domains. This approach was chosen to elicit rich accounts of “work as done,” uncover process breakdowns and tacit workarounds, and surface role-specific suggestions for more integrated, intelligence-ready operations. The reporting adheres to the Consolidated Criteria for Reporting Qualitative Research (COREQ) guidelines, where applicable (25).

### 2.2 Setting, participants, and sampling

The study was conducted in seven tertiary hospitals in Hunan, China. Hospitals were included based on two criteria: (1) tertiary hospital status, and (2) the deployment of core operational platforms (e.g., HIS, HRP/ERP, OA, SPD, and PACS). While the maturity and integration levels of these platforms differed across departments (e.g., some departments relied on manual reconciliation between systems for equipment, materials, or work orders, whereas others had these processes routinely linked across platforms), their widespread adoption enabled a consistent basis for analyzing cross-system operational workflows. We used purposive sampling to recruit key informants occupying roles with direct responsibility for equipment and resource management, including equipment management leaders and maintenance engineers, information/network center leadership and staff, ICU and ward nurse managers and nursing department leaders, general affairs/logistics leadership and warehouse officers, and procurement officers in the medical equipment division. Departmental introductions and snowballing facilitated recruitment; variation in seniority, tenure, and clinical context (e.g., ICU versus general wards) was sought to maximize diversity of perspectives. We continued sampling and analysis iteratively until data saturation was reached. In practice, the emergence of new codes began to plateau after the first 10 interviews. By approximately the twelfth interview, subsequent interviews yielded no substantively new codes, serving primarily to confirm and refine existing categories. Throughout data collection, we periodically reviewed whether additional interviews would likely provide substantively new insights relevant to the study’s aims. Recruitment was halted when further interviews were deemed to offer limited incremental information on the focal workflows and challenges.

### 2.3 Data collection

Data collection followed a semi-structured interview approach, with questions organized around key operational domains relevant to the study aims. Specifically, interviews covered (i) workflow and equipment lifecycle management, including daily responsibilities and end-to-end processes such as inspection, preventive maintenance, break/fix, calibration, procurement, acceptance, logistics, and approvals, as well as perceived pain points across the equipment lifecycle and the traceability of high-value consumables; (ii) IT systems and data management, focusing on experiences with core operational platforms—HIS, HRP/ERP, OA, and SPD—and related issues such as system usability, data silos, and duplicate data entry; (iii) operational capabilities and training, including needs for advanced functionalities (e.g., remote support, predictive alerts, executive dashboards, emergency dispatch, and compliance reminders), existing training practices, and barriers to adoption; and (iv) security, privacy, and emergency response, addressing data security and privacy considerations (including deployment models) as well as emergency response and incident handling. Question wording and sequencing were flexibly adapted to participants’ specific roles and operational contexts to elicit detailed accounts of “work as done” (Table 1). Interviews were conducted in Chinese, audio-recorded with informed consent, and transcribed verbatim. Field notes captured contextual observations and initial analytic insights (e.g., emerging codes, themes, and preliminary interpretations). For reporting, quotations were translated into English and attributed using broad role descriptors (e.g., clinical frontline staff, clinical management, technical/IT, logistics/administration) to preserve anonymity.

**Table 1:** General information of participants.

ID	Age	Education	Position	Work experience	Department group
N1	40–49	Bachelor	Management	21 + years	Logistics/technical
N2	40–49	Master	Clinical Management	21 + years	Logistics/technical
N3	30–39	Bachelor	Technical / IT	11–20 years	Information/IT
N4	30–39	Bachelor	Clinical (Frontline)	11–20 years	ICU/critical care
N5	30–39	Bachelor	Clinical (Frontline)	11–20 years	ICU/critical care
N6	30–39	Bachelor	Clinical (Frontline)	11–20 years	ICU/critical care
N7	40–49	Bachelor	Clinical (Frontline)	21 + years	ICU/critical care
N8	40–49	Bachelor	Clinical (Frontline)	21 + years	ICU/critical care
N9	50+	Master	Clinical Management	21 + years	Clinical administration
N10	40–49	Bachelor	Clinical Management	21 + years	Clinical administration
N11	30–39	Master	Clinical Management	11–20 years	Clinical administration
N12	40–49	Master	Management	21 + years	Clinical administration
N13	30–39	Master	Clinical Management	11–20 years	Clinical administration
N14	40–49	Bachelor	Administrative	21 + years	Administration
N15	20–29	Bachelor	Clinical (Frontline)	0–5 years	Administration
N16	30–39	Master	Technical / Logistics	6–10 years	Logistics/technical

### 2.4 Data analysis

Analysis followed a hybrid inductive–deductive strategy, implemented within a reflexive thematic analysis framework. This framework was selected because it allows analytic categories to be iteratively refined through engagement with the data, while remaining attentive to the researchers’ interpretive role and the organizational context of hospital operations. In the initial phase, two analysts independently conducted open coding on a subset of transcripts to surface in-vivo concepts and process markers, including approval bottlenecks, “black-hole” lending (informal borrowing of equipment or supplies without clear return or tracking), paper ledgers used as safety nets, and single-point failures in routine workflows; through iterative discussion and analytic memoing, these preliminary codes were consolidated into a shared codebook. The codebook was organized around seven overarching themes (e.g., equipment lifecycle management, logistics and materials, information systems, intelligent functions, training, data security, and cross-department collaboration), each representing a primary operational domain identified across the corpus, and further elaborated into 31 sub-themes; the deductive component of the analysis drew on these pre-identified operational domains to provide an initial analytic structure, while inductive coding captured emergent practices, workarounds, and role-specific interpretations described by participants. Focused coding and constant comparison were then applied to refine thematic boundaries, integrate perspectives across roles, and actively seek disconfirming cases; inductively derived codes were repeatedly compared across departments and mapped back onto the deductive framework to clarify relationships among sub-themes, with sub-theme frequency counts tracked as indicators of analytic salience to guide interpretation rather than as statistical measures. Stakeholder personas (e.g., equipment engineer, ICU head nurse, procurement officer) were used as an analytical lens to examine how responsibilities, information flows, and handoffs differed across roles, supporting the identification of interdependencies and coordination gaps that span departmental and professional boundaries.

### 2.5 Trustworthiness

To enhance trustworthiness, we employed multiple strategies grounded in established qualitative research criteria (26). Credibility was supported through triangulation across diverse roles involved in hospital operations, such as equipment engineers, ICU head nurses, procurement officers, and facilities or logistics staff, as well as through the use of rich verbatim quotations and the deliberate search for negative cases that challenged preliminary interpretations. Dependability was strengthened by maintaining an audit trail that documented the evolution of the codebook, key thematic decisions, and exemplar quotations. Additionally, reflexive memos were used to document analytic rationales and methodological decisions throughout the analysis process.

Confirmability was addressed by preserving these audit materials and by explicitly reflecting on the analysts’ professional backgrounds in systems and operations management. Such reflections were documented in reflexive memos, which noted how prior experience might sensitize the analysts to issues of system integration, coordination, and workflow efficiency, and how these assumptions were actively examined during analysis. Transferability was facilitated by providing detailed descriptions of the institutional context, the information systems landscape, and role responsibilities, enabling readers to assess the applicability of the findings to similar tertiary hospital settings.

### 2.6 Ethics statement

The study adhered to the ethical principles outlined in the Declaration of Helsinki and to institutional requirements for interview-based research. Institutional oversight (CSUCEEC-2025-008) was obtained through the hospital’s research governance process. All participants received an explanation of the study aims and procedures, provided informed consent before interview and recording, and were reminded that participation was voluntary and could be withdrawn at any time. Personal identifiers were removed during transcription; participant characteristics were minimized in tabular presentation, and only role descriptors are used in reporting to protect anonymity.

## 3 Results

### 3.1 Characteristics of participants

In the present study, 16 participants (N1–N16) were surveyed, with ages ranging from, with ages spanning multiple age groups, reflecting both early-career and senior professionals (Table 1). Educational attainment was predominantly undergraduate (bachelor’s degree; *n* = 11), with five holding master’s degrees. Participants had diverse roles across departments, including Nursing Administration (Directors and Deputy Directors), ICU/clinical nurse managers (Head Nurses), Equipment Management, Information Network Center, General Affairs, Infrastructure/Operations, and Medical Equipment/Procurement. Professional ranks spanned junior to senior levels (e.g., junior, engineer, charge nurse, associate senior, senior). Work experience spanned from early to advanced career stages, indicating a cohort with a broad range of professional experience.

### 3.2 Overview of themes

Reflexive thematic analysis identified seven interrelated themes comprising 28 sub-themes, which together delineate the structural conditions, constraints, and capability requirements shaping hospital operational management in tertiary Chinese hospitals. These themes encompass medical equipment lifecycle management, logistics and materials management, energy and facilities oversight, information system utilization, intelligent system requirements, training and organizational support, data security and privacy, as well as cross-departmental collaboration. Below, we elaborate on each theme and sub-theme in depth, incorporating illustrative quotations translated from Chinese to preserve meaning while maintaining anonymity (Table 2).

**Table 2:** Perceptions of participants in the interview.

Sub-theme	Example
Theme 1: Pain Points in Medical Equipment Management	
1.1: Medical equipment requires integrated, full lifecycle management from procurement to decommissioning, alongside the pressures of cross-process coordination	N2: For all our medical equipment, we handle end-to-end, full lifecycle management, from tracking orders and delivery to acceptance, maintenance, and service, and ultimately to decommissioning and replacement. N15: We really want to manage each asset with barcodes. After we scan a barcode, we can immediately see which department owns the asset and where it’s supposed to be.
1.2: Maintenance and repair must improve timeliness and professionalism to mitigate clinical risks arising from insufficient backup equipment	N2: A lot of our devices do not have backup units… For single-unit devices, repairs have to be fast and responsive. The service engineer’s skills need to be strong; otherwise, it’s hard to fix, and it just ends up taking longer. N16: Most of our repairs are urgent. We use a fast-track process where we fix as we go. We need to keep equipment running efficiently while also keeping the process compliant. N3: We later found that a fiber line had failed, which cut off the whole IT system. It was a Monday, and business was down for more than 20 min.
1.3: Utilization and cost-effectiveness should be evaluated through granular, objective data rather than experiential judgment	N1: We also examine cost-effectiveness, assessing whether the equipment is being used appropriately and delivering value after purchase. Our two primary metrics are uptime and cost–benefit. N14: Take this machine as an example: we consider how many patients it served, the revenue it generated, the number of repairs required, the total years in use, the extent of depreciation, the expenditures on repairs, and the associated staffing costs. When viewed together, these factors make the overall benefit readily apparent.
1.4: Cross-departmental allocation and tracking of equipment should be systematized to replace manual communication, thereby enhancing availability and transparency.	N4: I want a decision system that can tell me, at any time, how many devices are available for emergency redeployment across smaller hospitals. For example, which department has ventilators in the emergency pool. N7: I hope it can give us warnings and alerts.
Theme 2: Logistics and Materials Management	
2.1: Procurement processes should be anchored in standardized requirements and collaborative approvals to enable precise inquiry and negotiation.	N5: These days, we talk a lot about AI software. For what we need, AI could analyze and auto-generate some parameters to help departments clarify requirements. That’s really important. N7: Right now, we use the OA system to apply for procurement data, while procurement, ordering, delivery, and settlement are all handled in the SPD system. N10: Honestly, incomplete requirements are a big issue for procurement.
2.2: Inventory management requires broader coverage of systemic early-warning and automated replenishment while reducing the burden of manual stocktaking.	N4: You can set upper and lower stock limits. If I’m above the upper limit, I do not need to restock. If I drop below the safety stock, the system auto-alerts, creates a purchase order, and auto-places it with the vendor. N11: We conduct manual inventory counts, essentially once a month, with a major count every six months to a year. The monthly counts are smaller in scope. N16: Our general warehouse system has not been updated in more than ten years. Much of the work still relies on manual counting and tallying, and there are no inventory reminders.
2.3: High-value consumables must be governed by item-level unique identification with closed-loop traceability, ensuring real-time visibility of utilization flows.	N9: For high-value consumables, we use one-item-one-code so that we can track usage precisely. Scan the code, and it logs straight into the system, and we can see it in real time. The system is quite comprehensive.
2.4: Energy resources (water, electricity, gas) should be managed via tiered monitoring, trend analysis, and demand-based automated control to reduce waste.	N13: In the transitional seasons… it can lead to energy waste. N15: We want real-time data analysis, with year-over-year and month-over-month comparisons, plus automatic control. Ideally, we’d see energy use for each department.
Theme 3: Current Status and Challenges of Information Systems	
3.1: Existing OA/HRP/HIS/SPD systems, while improving efficiency, still exhibit limitations in adaptability and user experience.	N1: Our HRP system has a big market share nationwide… It’s just that some staff aren’t used to it. N6: Our OA system has basically gone paperless for approvals… but we are still lacking in newer smart/AI features.
3.2: Data silos across systems result in duplicate data entry and difficulties in cross-system data retrieval, necessitating interface interoperability.	N8: It’s not integrated with the operations platform… contracts, invoices, and acceptance documents all have to be uploaded and approved in multiple systems. N11: Our three systems do not talk to each other. We manage our procurement data; HIS manages consumption data; then the clinical team does a stock count and triggers replenishment.
3.3: System complexity and insufficient training elevate frontline users’ adoption barriers and adversely affect process execution.	N7: We need hands-on training for some systems… problems come up during real use, and then we fix them. N14: Online or offline? Definitely offline. Only face-to-face lets you ask questions and get answers right away. N16: On-site training works better. If there’s a problem, someone can come in person or guide us by phone. On-site plus lots of practice helps.
3.4: Manual records and fragmented ledgers increase the workload and error rate in data collection and analysis.	N5: We use the system for asset management to automatically record and manage things… but some items that generate costs still need manual entry. N9: Right now, we also keep a hospital-maintained ledger for instruments and equipment… It’s handwritten. I’d much rather have it all in a digital format.
Theme 4: Requirements and Suggestions for Intelligent Systems	
4.1: Equipment management requires remote expert support and integration with a knowledge base, leveraging historical records for fault prediction and self-diagnostic prompts.	N2; We need remote expert support, a maintenance knowledge base, access to repair history, and cross-department reminders, all of which should be integrated with the office system. N10: Can the machine self-check? Over time, what risks might grow as equipment ages?
4.2: Inventory management requires real-time stock alerts, dynamic distribution by department, and automatic ledger generation to reduce regulatory inspection workloads.	N3: If my stock goes below the lower limit, I want auto-alerts, auto-generated orders, and automatic POs sent to suppliers. N7: If there’s software for this, we’d like to use it, because monitoring and data collection are still pretty challenging.
4.3: Energy management necessitates terminal-facing real-time monitoring, anomaly alerts, trend analysis, and linkage with automated control strategies.	N14: We want real-time data analysis with YoY/MoM comparisons and automatic control.
4.4: Decision support should employ visualization, cross-device comparisons, and automated reporting to enable refined operational management.	N2: If it can present the data to us, we can analyze it and get meaningful benefit insights. N3: We’re exploring smarter solutions… even to the point where, based on certain patterns, it can suggest actions in time.
4.5: Emergency response requires systematized, rapid equipment localization and allocation, with workflow-driven, one-click activation for procedural support.	N8: If there’s an emergency somewhere… I want a decision system that shows how many devices are available for emergency redeployment. N12: If there’s a smart system, we’d like one-click rapid response, auto-generated task lists, and traceable workflows.
4.6: Compliance and safety require embedded capabilities for expiry reminders, automatic task generation, and cyclical inspection management.	N2: For next year’s inspections or metrology checks, there should be in-software reminders for due dates.
Theme 5: Training and Support	
5.1: Training for new systems should focus on key task operations and build advanced module competencies for engineers.	N1: We need systems that you can really practice on… train while using, keep practicing hands-on, and after a few rounds, you get the hang of it.
5.2: Training modalities should prioritize on-site demonstrations and learn-by-doing approaches, supplemented by microlearning videos and online materials.	N7: The IT team will provide training, including short videos with tips. That should take care of most issues. N9: On-site training works better. If there’s a problem, someone can come or guide us by phone. On-site plus more usage helps.
5.3: Training audiences should encompass clinical, managerial, and logistics roles, implemented via role-based stratification.	N5: For routine training, it’s best if each department has a fixed person responsible for handling operations.
Theme 6: Data Security and Privacy	
6.1: Data should be encrypted at the source and governed by role-based, tiered authorization to prevent unauthorized access and exfiltration.	N3: First, we need to improve our firewall setup… Second, tighten daily access control: based on role and level, you only get the permissions you need.
6.2: Core business functions should preferentially adopt on-premises deployment and dedicated communication channels to ensure availability and security.	N16: Why deploy locally? Put the system server inside the hospital. That way, it’s safe to use internally, and the data will not leave.
6.3: Maintenance and traceability data involving patient information should be de-identified and subject to rigorous compliance auditing.	N14: Adverse events include consumables and equipment… Every year, we have to file adverse event reports.
Theme 7: Cross-Departmental Collaboration	
7.1: Cross-departmental collaboration requires unified notification and coordination mechanisms to reduce information latency and risks associated with verbal transmission.	N11: We typically have a repair request system. At present, we use a maintenance system to submit repair requests, but it operates as a separate, independent system. N15: If an urgent issue arises, we follow the hospital and departmental emergency plans and coordinate the response, usually by phone.
7.2: Process optimization should shorten approval chains, solidify responsibility boundaries, and leverage system logs to support accountability.	N1: If a repair costs over 10,000, we have to go through negotiation/tendering… and sign a contract. So that’s basically our process.

### 3.3 Theme 1: Pain points in medical equipment lifecycle management

#### 3.3.1 Sub-theme 1.1: end-to-end lifecycle scope and coordination burden

Participants consistently described device management as a cradle-to-grave responsibility that spans specification, procurement, acceptance, commissioning, break/fix and preventive maintenance, calibration/metrology, replacement, and decommissioning. This broad scope of equipment lifecycle management necessitates frequent handoffs among multiple departments, including the equipment office, logistics, finance, and clinical units. In practice, these cross-departmental interactions are further complicated by fragmented identifiers and records, which increase coordination effort and hinder information continuity. One logistics/technical staff member (N02) participant summarized the scope: “We manage the entire lifecycle, including order follow-up, acceptance, maintenance and upkeep, and ultimately scrapping and replacement.” The need for identity continuity and location binding was repeatedly emphasized: “We still hope to manage each asset via barcodes; once we scan the code, we should know the owning department and its original location” (Administrative staff, N15). In the absence of a unified asset backbone, participants reported duplicate data capture and “paper ledger fallbacks,” which increase cognitive load and impede timely reconciliation during audits and incident reviews.

#### 3.3.2 Sub-theme 1.2: maintenance responsiveness and professional depth under limited backups

Hospitals’ lean backup pools heighten the risk of single-point failures, shifting the burden onto maintenance responsiveness and engineer skill depth. As a logistics/technical informant (N02) noted, “Many devices have no spares… for single-unit equipment, response time and the engineer’s professional skill must be strong; otherwise resolution is delayed.” Operational adaptations, exemplified by “emergency repairs via a quick channel, in which equipment is fixed while still in use” (Technical/logistics staff, N16), reflect a necessary yet fragile strategy to sustain throughput while adhering to procedures. Infrastructure failures magnify downtime externalities: “A single fiber fault broke our information systems; being a Monday, business stalled for 20-plus minutes” (Technical/IT staff, N03). These accounts highlight the dependence on vendor schedules, long lead times for parts, and the limited in-house expertise for specialized modalities, which together extend mean time to repair and propagate delays into clinical services.

#### 3.3.3 Sub-theme 1.3: utilization and cost-effectiveness require objective, device-level analytics

Decision-makers articulated a preference for evidence-based, device-level utilization and Return On Investment (ROI) analytics over experience-based judgments. One participant from a management role (N01) stated, “We care most about uptime and cost-effectiveness, asking whether the device ultimately delivered its expected value after purchase.” Desired measures included case volume, uptime, repair counts, depreciation, maintenance cost, and staffing inputs: “How many patients the machine handled and the revenue it generated, the number of repairs, the years in use, the depreciation profile, the repair expenditures, and the associated staffing requirements together reveal its overall benefit.” (Technical/logistics staff, N14). Current gaps, including manual logs, scattered information sources, and inconsistent denominators, limit the precision of replacement planning, budget allocation, and contract renegotiation, with several respondents acknowledging reliance on “best guesses” in leadership briefings.

#### 3.3.4 Sub-theme 1.4: systematized dispatch and traceability to replace *ad hoc* coordination

*Ad hoc* lending and phone-based coordination can obscure asset availability and accountability, particularly in emergency scenarios (e.g., ventilator dispatch). Leaders called for a systemized dispatch layer with live readiness and location status: “I want a decision system that shows how many ventilators are in the emergency dispatch pool, and exactly where they are” (Technical/logistics staff, N04). Clinical units requested anticipatory warnings tied to condition indicators: “We hope it can give some early warnings” (Clinical frontline staff, critical care, N07). Absent such capabilities, participants described cross-unit competition for scarce spares, unclear liability in case of damage, and *post hoc* reconstruction of movement histories that hinder auditability and continuous improvement.

### 3.4 Theme 2: logistics and materials management

#### 3.4.1 Sub-theme 2.1: standardized requirement specification and collaborative approvals

Underspecified requests and complex approval processes often hinder procurement. A logistics/administrative staff member (N10) observed, “Insufficient requirement information is a major procurement problem,” which compromises market comparison and post-delivery acceptance criteria. Participants suggested Artificial Intelligence (AI)-assisted parameter templating to strengthen completeness and comparability: “Based on what we need, [AI] can automatically generate parameters, clarifying requirements” (Logistics/administrative staff, N05). Process-wise, dual-track tools create friction: “Applications are via OA, but purchasing, ordering, delivery, and settlement are via SPD” (Logistics/administrative staff, N07), resulting in duplicated uploads and status checks. These seams elongate cycle times and increase rework when specifications are challenged downstream.

#### 3.4.2 Sub-theme 2.2: inventory coverage, proactive alerts, and auto-replenishment

Inventory management remains uneven, with several stores lacking system prompts and relying on monthly or semiannual manual counts: “Our general affairs storeroom has not upgraded systems for over a decade; much is still manual counting and statistics, with no inventory prompts” (Administrative staff, N16). Respondents advocated min–max policies with automated order generation to mitigate stockouts and reduce manual workload: “Set upper and lower limits… if below the lower bound, auto-alert, generate system orders, and auto-place with suppliers” (Logistics, N04). The absence of dynamic, per-department distribution views hinders intra-hospital rebalancing, particularly during demand surges.

#### 3.4.3 Sub-theme 2.3: one-item-one-code traceability for high-value consumables

For high-value consumables, participants endorsed closed-loop, item-level traceability via unique identifiers and bedside scanning: “For high-value consumables, we use one-item-one-code; scan the code and the information records in the system, which we can view in real time” (Clinical management staff, N09). Reliable binding at the point of care was viewed as essential to satisfy audits, manage recalls, and clarify charge capture, while also reducing the burdens of retrospective reconciliation. Breakdowns were attributed to inconsistent scanning practice, device availability, and integration gaps with clinical documentation.

#### 3.4.4 Sub-theme 2.4: tiered energy monitoring, trend analytics, and automated control

Facilities leaders reported energy waste during transitional seasons due to coarse-grained HVAC control and limited sub-metering. “During transitional seasons… it causes energy waste” (Clinical management staff, N13). Desired capabilities included real-time monitoring, comparative analytics across Year-over-Year (YoY) and Month-over-Month (MoM) timeframes, anomaly detection, and policy-driven auto-tuning down to departmental zones: “Ideally, understand each department’s energy use” (Clinical frontline staff, N15). Absent these, leak/surge detection and zone optimization are reactive rather than anticipatory, with cost and comfort implications.

### 3.5 Theme 3: information systems usage and challenges

#### 3.5.1 Sub-theme 3.1: efficiency gains alongside usability and fit gaps

While OA/HRP/HIS/SPD deployments have improved standardization and reduced paper, fit-for-use remains inconsistent across roles and digital literacy levels. “Our HRP has significant market share… but some staff are not used to it” (Management staff, N01). Similarly, the OA platform “is nearly paperless for approvals, but there are still shortcomings in smart/intelligent features” (Clinical frontline staff, N06). These gaps manifest as longer task completion times, help-seeking via informal channels, and a return to spreadsheets and paper notes. Across themes, this return to spreadsheets and paper notes illustrates how usability and fit gaps translate into fragmented “work-as-done” records, weakening organization-wide situational awareness and slowing coordinated action.

#### 3.5.2 Sub-theme 3.2: data silos, duplicate entry, and cross-system reconciliation

Participants frequently cited the absence of interoperable interfaces across procurement, contract, acceptance, and consumption domains: “It’s not integrated with the operations platform… contracts, invoices, acceptance must be re-uploaded and approved across systems” (Clinical frontline staff, N08). A clinical management staff member (N11) informant added, “Our three systems do not interconnect; procurement manages procurement data, the HIS manages consumption, and clinical staff perform restocking after manual counts.” These seams increase error risk, obscure provenance, and undermine near-real-time situational awareness for leaders.

#### 3.5.3 Sub-theme 3.3: system complexity and the need for hands-on, iterative training

This sub-theme addresses training needs that arise directly from system complexity and the infrequent occurrence of specific operational tasks during routine use, rather than from formal training arrangements. Frontline users emphasized that complexity and low frequency of specific tasks necessitate practical, iterative learning: “We require practical operation… train while using, solve problems encountered during use” (Clinical frontline staff, N07). Preference for in-person modalities was pronounced: “Offline training is preferable, as face-to-face instruction allows timely questions and answers.” (Management staff, N14); “On-site training works better, with immediate phone or on-site guidance” (Technical/logistics staff, N16). These preferences indicate the need for microlearning integrated into workflows, rather than one-off classroom sessions.

#### 3.5.4 Sub-theme 3.4: manual records and scattered ledgers as safety nets

Despite digital systems, non-integrated cost events and operational exceptions still require manual input: “Some items that incur costs still require manual input” (Clinical frontline staff, N05). Units often maintain parallel ledgers, sometimes handwritten, as “safety nets.” “We maintain an internal instrument ledger… currently handwritten; we prefer a data-driven form” (Clinical management staff, N09). These practices, while adaptive, introduce transcription errors and complicate auditing. Meanwhile, these manual records function as informal safeguards against system gaps, but they also fragment data provenance, increase reconciliation effort, and ultimately limit hospitals’ ability to obtain timely, organization-wide operational visibility.

### 3.6 Theme 4: Needs and suggestions for intelligent systems

#### 3.6.1 Sub-theme 4.1: remote expert support, maintenance knowledge bases, and predictive/self-test cues

Building on the fragmentation, usability, and coordination constraints described in Theme 3, stakeholders requested technician-centric tooling that integrates remote expert access, a searchable maintenance knowledge base, and device self-checks with risk alerts. “Remote expert support, maintenance knowledge base, and access to historical records… with collaborative reminders” (Clinical management staff, N02). Anticipatory diagnostics were specifically desired: “Can the machine self-check… and warn about risks that increase with age?” (Clinical management staff, N10). These capabilities were framed as practical enablers for faster fault resolution and skill amplification, particularly for high-impact assets.

#### 3.6.2 Sub-theme 4.2: inventory dashboards with real-time alerts, dynamic distribution, and auto-ledgering

Participants envisioned dashboards that couple threshold-based alerts with per-department distribution maps and automatic ledger/report generation to ease regulatory inspections. “Below the lower bound, auto-alert and auto-order to the supplier” (Technical/IT staff, N03). “For monitoring and data collection, software is still needed because it’s difficult” (Clinical frontline staff, N07), indicating the importance of scan-to-act interfaces and minimal click workflows.

#### 3.6.3 Sub-theme 4.3: energy monitoring with anomaly detection and policy automation

Facilities leaders requested terminal-level instrumentation, anomaly detection, comparative analytics, and strategy linkage that automates control changes. “Real-time analytics with YoY/MoM comparisons and automatic control” (Management staff, N14). Such features were positioned not merely as visualization, but as levers to move from reactive investigation to proactive energy stewardship.

#### 3.6.4 Sub-theme 4.4: decision support through visualization, benchmarking, and automated reporting

Managers sought standardized KPIs and visual dashboards to support like-for-like comparisons across device classes and departments. “The system should present data so we can analyze and achieve benefit analysis” (Clinical management staff, N02). IT leaders extended this aspiration to recommendation generation: “We’re exploring further intelligence… to give timely plans based on characteristics” (Technical/IT staff, N03). Routine, automated reports were deemed necessary to alleviate the burdens of manual compilation.

#### 3.6.5 Sub-theme 4.5: emergency response with rapid device location, readiness, and workflow activation

Emergency orchestration was a recurring need: “When emergencies occur… I want a decision system showing how many devices are available for dispatch” (Clinical frontline staff, N08). Clinical management staff (N12) emphasized the need for a fast start and traceability: “If there is a smart system, we hope it can start quickly, auto-generate task lists, and keep processes traceable.” Participants highlighted the value of live readiness (e.g., battery status, calibration) and time-to-bedside estimates to compress response latency.

#### 3.6.6 Sub-theme 4.6: embedded compliance and expiry management

Respondents called for system-level prompts and auto-tasking for inspections, calibration, and metrology: “Annual inspections and metrology should trigger expiry reminders in software” (Clinical management staff, N02). Embedding these into routine workflows with accountable owners and escalations was viewed as essential to prevent lapses that only surface during audits.

### 3.7 Theme 5: training and support

In contrast to Sub-theme 3.3, which reflects training needs that emerge during system use, this theme focuses on training and support as an organizational capability, encompassing institutional arrangements, delivery modes, role-based coverage, and long-term skill development.

#### 3.7.1 Sub-theme 5.1: mission-critical focus and advanced modules for engineering capability

Participants advocated training that prioritizes high-stakes workflows and deepens engineering proficiency through repeated, situated practice: “We must practice… training while using, and after several real operations, one becomes proficient” (Management staff, N01). This approach was contrasted with one-time classroom sessions that fail to transfer under pressure.

#### 3.7.2 Sub-theme 5.2: on-site demonstrations, learn-by-doing, and micro-learning assets

A blended model was preferred, incorporating on-ward demonstrations, immediate phone or in-person troubleshooting, and short videos covering specific tips. “The information department provides training, including short videos with tips, which can solve issues” (Clinical frontline staff, N07). “On-site training works better; immediate guidance by phone or in person is more effective” (Clinical management staff, N09). These modalities were viewed as inclusive of night and weekend staff.

#### 3.7.3 Sub-theme 5.3: role-wide coverage with tiered curricula and designated super-users

Coverage should include clinical, managerial, and logistics staff, with fixed departmental contacts to sustain capability and reduce support latency: “At least each department should have a designated operator; fixed personnel are best” (Clinical frontline staff, N05). Tiered curricula (basic, advanced, administrator) were proposed to align with role responsibilities and turnover.

### 3.8 Theme 6: data security and privacy

#### 3.8.1 Sub-theme 6.1: encryption at source and least-privilege, role-based access

Security stakeholders emphasized defense in depth: “Improve the firewall… and strengthen routine permission management by granting only role-appropriate access.” (Technical/IT staff, N03). Participants called for auditable changes, privileged access management, and continuous monitoring to deter misuse and exfiltration.

#### 3.8.2 Sub-theme 6.2: on-premises deployment and dedicated channels for core operations

A strong preference for on-premises hosting aimed to ensure availability, sovereignty, and control: “Localize deployment; put servers inside the hospital so data does not leave and internal use is secure” (Technical/logistics staff, N16). Dedicated network channels and segmentation were viewed as necessary to isolate operational systems from public networks and reduce the attack surface.

#### 3.8.3 Sub-theme 6.3: de-identification and compliant auditing for patient-related maintenance/traceability

Participants acknowledged that maintenance, incident, and traceability data may intersect with patient identifiers and, therefore, require de-identification and rigorous audit trails. As one management staff member (N14) noted, “Adverse events include both consumables and devices, and we are required to report them annually,” underscoring the need for compliant logging, retention, and role-based visibility policies.

### 3.9 Theme 7: cross-department collaboration

#### 3.9.1 Sub-theme 7.1: unified notification and coordination mechanisms to replace parallel channels

Multiple, parallel reporting and communication tools create latency, duplication, and loss of institutional memory. “We have one repair system, and our maintenance system is another, entirely separate repair system.” (Clinical management staff, N11). Incident coordination remains largely telephone-based: “For emergencies… generally by phone” (Clinical frontline staff, N15). This reliance on telephone-based coordination is not merely a communication preference; it signals a broader operational challenge—namely, the absence of a traceable, standardized, system-integrated workflow that can sustain coordination quality and accountability under time pressure. Participants advocated a single, traceable channel with read receipts, escalation paths, and integration to asset and inventory records.

#### 3.9.2 Sub-theme 7.2: streamlined approvals, clear accountability, and system trace for governance

Approval chains, especially for high-value repairs and purchases, were described as lengthy and rigid: “Repairs over 10,000 Chinese yuan (RMB) must go through negotiation or tendering and contract signing” (Management staff, N01). Clarifying role boundaries and embedding digital traces were proposed to expedite action without compromising accountability. An IT leader’s closing remark, “That is our process,” captured the institutionalization of current practices and the challenge of change, reinforcing the need for governance mechanisms that codify faster pathways under defined risk thresholds.

## 4 Discussion

This multi-stakeholder qualitative study reveals a consistent sociotechnical pattern: mission-critical equipment, inventory, and facilities management are constrained by fragmented data flows, uneven process maturity, and limited real-time visibility. Across clinical, technical, and administrative roles, participants described doing “work around the system” with spreadsheets, messaging apps, and phone calls because core platforms (HIS, HRP, OA, SPD, and facilities controls) are poorly integrated and cumbersome to use (27). These workarounds keep services running but are brittle, unauditable, and slow, particularly when failures occur in single-point-of-failure devices or when urgent device redeployment is needed. The study adds granularity to prior reports of hospital data silos and maintenance backlogs by tracing how problems compound across the full lifecycle, from underspecified procurement requests, to scattered contracts and certifications, to manual utilization logs, to *ad hoc* lending and unclear liability (28). Importantly, participants did not request an abstract “AI” but rather concrete capabilities that reduce clicks, eliminate duplicate entries, provide trustworthy status and history, and deliver role-specific, timely prompts. The reliance on improvised, person-to-person communication channels also reflects a form of bonding social capital that enables local coping in the short term but lacks the scalability and reliability needed for sustained organizational resilience during major system shocks. Taken together, these patterns point to a set of underlying mechanisms—fragmented governance, incremental system deployment, and the displacement of coordination work onto individuals—that explain why operational challenges recur across equipment, inventory, physical facilities, and information systems.

Taken together, the findings suggest an intelligence-readiness agenda with several interdependent priorities. First, hospitals require a trustworthy and unified asset backbone. Unique identifiers must bind procurement, contract terms, commissioning dates, maintenance and calibration histories, current locations, and decommissioning (29). Without this spine, analytics or automation layers perpetuate mismatches that force manual reconciliation. Second, maintenance workflows should transition from delayed, vendor-dependent responses to faster, skill-amplified operations. Scan-to-report must be ubiquitous; remote expert guidance and model-specific knowledge bases should be readily available; and early-warning cues derived from usage hours, error codes, and basic telemetry should preempt failures (30). Third, inventory control should be consistent and visible. Thresholding and auto-replenishment reduce stockouts; dynamic distribution views let users see where items actually are by ward or OR; and one-item-one-patient traceability for high-value consumables must be reliable at the bedside, not reconstructed after the fact (31). Fourth, facilities and energy management should be real-time and mobile. Sub-metered water, electricity, and HVAC data need to be unified into dashboards that detect anomalies and enable zone-level optimization during transitional seasons (32). Fifth, decision support must be role-tuned and standardized. Executives require comparable views across device classes with automated reports; ICU and OR teams need rapid device dispatch tools with status-of-charge and time-to-bedside estimates; and compliance managers need systematic tracking of calibrations and certifications tied to accountable owners and escalation paths (33, 34). Collectively, these capabilities strengthen hospitals’ ability to anticipate, absorb, and adapt to disruptions—key dimensions of organizational resilience that determine whether essential services can be maintained for surrounding communities during crises.

Translating these priorities into a practical roadmap requires sequencing, integration discipline, and human-centered design. Each phase of the proposed roadmap corresponds to a specific cluster of challenges identified in the Results. Phase 1 addresses foundational deficits in asset lifecycle integration, logistics visibility, information system silos, and cross-departmental coordination (Themes 1, 2, 3, and 7). Phase 2 targets limitations in responsiveness and predictability—such as delayed maintenance, emergency device availability, and *ad hoc* dispatch—through predictive maintenance and coordinated resource mobilization (Themes 1, 2, and 4). Phase 3 extends these capabilities toward sustained optimization, governance, and learning by institutionalizing training and support, standardizing decisions, and managing data security and privacy (Themes 5 and 6). A credible first phase invests in data plumbing and event orchestration. This includes cleaning and linking the asset registry across systems, instrumenting scan-to-report maintenance, wiring event-driven reminders for contract renewals, certificate expirations, scheduled service, inventory thresholds, and facilities anomalies, and implementing closed-loop communications with read receipts and escalation across departments (35, 36). In the Chinese tertiary hospital context, this “data plumbing first” sequencing is also policy-relevant because DRG/DIP reforms intensify scrutiny of utilization, cost-effectiveness, and traceability, which fragmented and manually reconciled records cannot reliably support. In parallel, interfaces should minimize cognitive load through contextual prefill and scan-to-act patterns, while training shifts from lengthy manuals to micro-learning at the point of use, for example, through QR codes on devices that launch two-minute “how-to” videos, complemented by on-the-job demonstrations that also reach night and weekend staff (37). Security and privacy cannot be bolted on later. Encryption at rest and in transit, least-privilege permissions, privileged access management, on-premises or private deployments for core operational data, de-identification of patient context in non-clinical views, and full auditability are prerequisites for sustained trust and resilience (38). This emphasis on on-premises or private deployment should be interpreted within the context of the Chinese tertiary hospital setting described earlier, where data sovereignty expectations and lessons from external service outages shape feasible implementation pathways. By institutionalizing reliable information flows and enforceable responsibilities, such foundations also reduce dependence on informal workarounds, shifting resilience from individual heroics toward collective, system-level capacity.

Within this staged modernization, digital twin technology offers a targeted approach to addressing the identified deficits in the results, including limited visibility, poor predictability, and complex coordination. This potential, however, depends on judicious deployment and a solid foundation of integration and governance (39). Digital twin technology refers to a living, virtual model of a physical asset, process, or environment that stays synchronized with its real-world counterpart via bidirectional data flows (40). It fuses telemetry (e.g., sensors, logs, error codes), configuration and master data, maintenance and utilization history, and contextual signals (location, environment, workload) into a governable, queryable representation that can simulate states, predict outcomes, and drive automated or human-in-the-loop actions (41). Because a twin consolidates fragmented data into a single source of operational truth, provides forward-looking predictions rather than retrospective reports, and orchestrates responses across roles and systems, it directly addresses the observed gaps of visibility, predictability, and coordination (42). In practice, twins reduce manual reconciliation, expose emerging risks early, standardize decision logic, and enable closed-loop control, making them a pragmatic bridge from today’s brittle workarounds to resilient, proactive operations (43).

At the asset level, virtual representations of critical devices, such as ventilators and imaging modalities, can synchronize continuously with real-world state signals (usage hours, alarms, component temperatures, and battery cycles) (44). Coupled with a curated maintenance knowledge base and histories of prior faults and fixes, these twins can surface early-warning indicators for component wear and failure modes, generate actionable work orders, and coordinate spares and vendor engagement. In settings with few or no backups, such predictive maintenance shifts operations from “firefighting” to prevention, directly mitigating the downtime risks described by engineers and ICU leaders (45). Such shifts are significant in crisis conditions, where single-device failures can cascade into service bottlenecks that undermine hospitals’ ability to provide continuous and equitable care.

At the process level, twins of key clinical and operational flows, such as operating room device readiness and turnover, emergency device dispatch, and imaging throughput, can integrate location data, readiness information (including battery and calibration status), and routing data to present a live picture of “which device is functional, where it is, and how long it will take to reach the bedside.” (46). This closes the gap between need and supply during crises and reduces the time-consuming, error-prone communication loops that participants reported. Process twins also enable fair, like-for-like benchmarking across similar devices and services on uptime, maintenance cost, energy consumption, and output proxies, informing budgeting, replacement, and staffing decisions (47). By improving horizontal (bridging) coordination across departments and vertical (linking) coordination between frontline teams and administrators, process-level twins strengthen the forms of social capital that facilitate collective action under stress.

Materials and traceability layers can utilize digital twin concepts to model the lifecycle of high-value consumables, from inbound receipt through distribution to bedside binding, procedure use, and reconciliation (48). When binding occurs reliably at the bedside via scanning and the lifecycle is represented end-to-end, audit-ready traceability emerges as a byproduct of work rather than a separate documentation task, thereby decreasing error rates and dispute potential. On the physical facilities side, building and zone-level twins can consolidate sub-metered streams for water, electricity, and HVAC, detect leaks and surges, and apply policy-driven zone adjustments that eliminate simultaneous overcooling and overheating during shoulder seasons (49). For facilities leaders who currently lack mobile, real-time visibility, this can shift the posture from reactive investigation to proactive management.

The feasibility constraints voiced by participants established necessary guardrails for introducing such capabilities. Integration debt and data silos necessitate interoperable identifiers and event streams across HIS, HRP, OA, SPD, PACS, and physical facilities systems before high-fidelity modeling can add value. Security imperatives recommend on-premises or private deployments for core operational data, strict encryption, least-privileged access, and rigorous de-identification, especially in contexts where non-clinical users access operational views (50). Retrofit economics suggest focusing first on high-impact, telemetry-ready assets and domains while planning for the selective modernization of legacy equipment that lacks interfaces (51). Human factors caution against feature bloat: insights should be presented as role-specific, actionable suggestions rather than raw technical dashboards, and learning should come in short, scenario-based assets that fit into real workflows (37).

A pragmatic adoption roadmap derived from the participants’ priorities could therefore proceed in three phases. The first phase delivers data foundations and quick wins, including unifying the asset registry, deploying scan-to-report functionality, automating compliance reminders and logs, implementing threshold-based inventory control with dynamic distribution views, launching mobile energy dashboards with basic anomaly detection, and establishing closed-loop notifications across departments. The second phase adds prediction and coordination, enabling predictive maintenance for a prioritized cohort, such as ICU ventilators, OR anesthesia and monitoring clusters, or imaging modalities using available telemetry. It also involves rolling out emergency device dispatch with live readiness and routing, as well as standardizing executive dashboards for cross-device benchmarking with automated monthly reports. The third phase expands to process-level optimization, focusing on model and improving OR turnover, imaging throughput, and zone-level energy management.

Additionally, it evaluates retrofit versus replacement pathways to broaden telemetry coverage cost-effectively. Throughout, governance should codify interface standards (data semantics, sampling cadence, and protocols), model validation (alert accuracy and explainability), access segmentation (role-based minimum visibility), resilience (offline fallbacks and disaster recovery), and continuous improvement (A/B testing and metric dashboards) (52). By aligning technical modernization with governance, equity, and human-centered safeguards, such a roadmap reinforces not only operational performance but also hospitals’ role as stability anchors within their surrounding communities during crisis conditions.

Although we distilled a comprehensive set of concrete design requirements from stakeholder input, their essence can be captured succinctly. Hospitals articulated a need for an integrated and coherent set of digital capabilities to support day-to-day operations and governance. At the asset and maintenance level, this included a unified and auditable asset ledger, event-driven reminders and workflows, technician-friendly mobile tools with remote guidance, a searchable maintenance knowledge base, and predictive maintenance for high-impact equipment, alongside real-time visibility into device readiness, location, and battery health to enable rapid dispatch during emergencies. From a control and governance perspective, respondents emphasized the importance of strict role-based access with de-identified patient context, bedside binding and reconciliation for high-value consumables, inventory thresholding supported by dynamic distribution views, and digitally managed lending with clear liability attribution. Procurement and administrative processes were expected to benefit from AI-assisted specification drafting, streamlined approval pathways, and compliance dashboards.

Meanwhile, energy and facilities management required integrated dashboards that support anomaly detection, zone-level auto-tuning, standardized benchmarking, and automated reporting. Beyond technical functions, participants emphasized the importance of closed-loop communication mechanisms, incentive structures for interdepartmental collaboration, and continuous capacity building through microlearning and on-the-job demonstrations. Security-by-design was consistently regarded as a foundational requirement across all system capabilities (53). Implemented as a coherent program, these capabilities answer the lived problems that surfaced in the interviews and create a runway for higher-order modeling and optimization. More broadly, these requirements illustrate how sociotechnical integration—when aligned with frontline realities—can cultivate the information reliability, trust, and coordinated action that underpin social cohesion and community-level resilience.

The study has strengths and limitations. Its strengths include triangulation across roles and departments, rich operational detail anchored in verbatim accounts, and the translation of qualitative insights into a staged, vendor-agnostic implementation blueprint. The study was conducted across seven tertiary hospitals within a single provincial context in China, which may limit its generalizability to other regions or healthcare systems. The salience counts attached to sub-themes are qualitative markers rather than quantitative estimates. We did not evaluate a deployed platform; therefore, the proposed benefits are theoretically grounded but untested in this context.

Additionally, nursing staff were numerically overrepresented, reflecting their central role in equipment use, incident reporting, and coordination in both daily operations and emergency contexts. This sampling structure may have influenced the salience of specific issues, and findings should be interpreted with attention to this role distribution. Future mixed-methods research could pilot phased implementations in selected domains and quantitatively assess operational and resilience-related outcomes. Meanwhile, future mixed-methods research should pilot phased deployments in prioritized domains (e.g., ventilators and OR device clusters) and measure impacts on downtime, mean time to repair, emergency response latency, compliance lapses, energy waste, and user burden. Economic analyses comparing retrofit and replacement strategies can inform investment decisions, and procurement policies can be updated to include “telemetry-ready” and “interface-ready” requirements, thereby reducing future integration debt. Further research should also examine how intelligence-ready infrastructure reshapes the relational and institutional dynamics—such as trust, coordination, and perceived reliability—through which hospitals support community resilience in crisis settings.

## 5 Conclusion

In summary, hospitals are ready for pragmatic, secure, and role-tuned intelligent capabilities that integrate with existing platforms, minimize duplicate entry, and deliver actionable value where it matters most. By sequencing efforts across data integration and event orchestration, human-centered user experience with micro-learning, and security-by-design practices, organizations can establish a stable operational foundation. Building on this foundation, progressive automation can be selectively introduced to improve efficiency and consistency. Where telemetry quality and expected returns justify the investment, high-fidelity operational modeling further enables reductions in downtime, faster emergency response, stronger compliance, reduced waste, and the recovery of time and cognitive bandwidth for clinical and engineering teams. Digital twin approaches, applied judiciously within this public health governance and integration context, are particularly well-suited to restoring visibility, enabling prediction, and coordinating action across high-impact assets and processes.

When viewed through the lens of public health organizational and community resilience, these intelligence-ready capabilities function not merely as efficiency measures but as foundational infrastructures that enhance hospitals’ ability to maintain continuity, responsiveness, and equitable access to care during disruptions. By improving information reliability, strengthening cross-departmental public health coordination, and reducing dependence on informal workarounds, an intelligence-ready hospital becomes a more stable and adaptive node in the broader health system, supporting community well-being in both routine and crisis public health settings.

## Data Availability

The original contributions presented in the study are included in the article/supplementary material, further inquiries can be directed to the corresponding author.
